# Structural and Dynamical Insight into PPARγ Antagonism: *In Silico* Study of the Ligand-Receptor Interactions of Non-Covalent Antagonists

**DOI:** 10.3390/ijms160715405

**Published:** 2015-07-08

**Authors:** Filip Fratev, Ivanka Tsakovska, Merilin Al Sharif, Elina Mihaylova, Ilza Pajeva

**Affiliations:** 1 Institute of Biophysics and Biomedical Engineering, Bulgarian Academy of Sciences, 1113 Sofia, Bulgaria; E-Mails: ITsakovska@biomed.bas.bg (I.T.); merilin.al@biomed.bas.bg (M.A.S.); 2 Micar21 Ltd., 1407 Sofia, Bulgaria; E-Mail: mihaylova@micar21.com

**Keywords:** PPARγ, antagonism, helix 12, nuclear receptors, molecular dynamics

## Abstract

The structural and dynamical properties of the peroxisome proliferator-activated receptor γ (PPARγ) nuclear receptor have been broadly studied in its agonist state but little is known about the key features required for the receptor antagonistic activity. Here we report a series of molecular dynamics (MD) simulations in combination with free energy estimation of the recently discovered class of non-covalent PPARγ antagonists. Their binding modes and dynamical behavior are described in details. Two key interactions have been detected within the cavity between helices H3, H11 and the activation helix H12, as well as with H12. The strength of the ligand-amino acid residues interactions has been analyzed in relation to the specificity of the ligand dynamical and antagonistic features. According to our results, the PPARγ activation helix does not undergo dramatic conformational changes, as seen in other nuclear receptors, but rather perturbations that occur through a significant ligand-induced reshaping of the ligand-receptor and the receptor-coactivator binding pockets. The H12 residue Tyr473 and the charge clamp residue Glu471 play a central role for the receptor transformations. Our results also demonstrate that MD can be a helpful tool for the compound phenotype characterization (full agonists, partial agonists or antagonists) when insufficient experimental data are available.

## 1. Introduction

A lot of data are available about the dynamics of peroxisome proliferator-activated receptor γ (PPARγ) and its agonists, collected by several experimental techniques such as hydrogen deuterium exchange (HDX) mass spectrometry, NMR and fluorescence anisotropy [1,2,3,4,5]. A detailed binding mode of this class of compounds has also been revealed by many X-ray structures deposed in the Protein Data Bank (PDB) [6]. A new class of non-covalent PPARγ antagonists sharing a similar skeleton to that of some known partial agonists has recently been reported [7]. Little is known about PPARγ antagonism and this information is based only on structural data for the covalently bound antagonists [8,9]. However, information is still missing about both the binding mode and the structural changes and dynamics of the non-covalent PPARγ antagonists. It is also unknown how the activation helix 12 (H12) is affected by the antagonists and how antagonists compare to partial agonists. Some of the partial agonists exhibit very low agonistic activity being closer to antagonists, rather than to agonists and their phenotype is sometimes difficult to describe by traditional experimental approaches. Thus, studies that reveal the potential binding mode and dynamics of these compounds and identify the link between the dynamics of partial agonists and full antagonists are required. Such studies can also contribute to a deeper understanding of the PPARγ dynamics.

The commonly adopted conception about the dynamics of the PPARγ full agonists relies on experimental data and suggests that they stabilize the H12 position, compared to the apo protein form, through interactions with the residue Tyr473 in particular. Other parts of the ligand-binding domain (LBD), such as H11 and H3, are also considered to play a role in the agonistic conformation of the receptor [5]. However, the observed conformational changes in H12 seem to be much smaller compared to other nuclear receptors, as, for example, it has been demonstrated for the estrogen receptor α (ERα) [10,11] and additionally by employing accelerated molecular dynamics (MD) [12]. The dynamics of partial and full agonist modes of action have been revealed in a series of studies [1,2,3,4]. It has been pointed out that these compounds not only affect H12 but also significantly alter the H3, H4 and β1–4-sheets region [1,2]. Further, it has been shown that some partial agonists can also bind in multiple modes and even in two different parts of the binding site simultaneously [1,13].

Very recently, a new class of non-covalent antagonists has been discovered, based on the skeleton structure of partial agonists [7]. The original hypothesis for the design of this class of antagonists is that if an additional, more rigid and bulky non-polar substituent is introduced in the region where the partial agonists interact with H12, in particular with Tyr473, this would result in a full antagonistic feature. However, the binding mode and the molecular mechanism of their action have not been specified yet. Moreover, the available experimental data do not allow to relate the ligands to a certain phenotypic class (partial agonists or antagonists) and to rank them according to their partial agonistic activity.

In the present work, which is the first part of our extensive MD studies of the PPARγ antagonism, we report a series of MD simulations of PPARγ complexes. Based on the results, we have proposed the binding modes of selected partial agonists and antagonists, as well as estimated their total free energy of binding and the binding energies to PPARγ residues involved in interactions. The phenotypic nature of the ligands has been explained on the basis of their structural differences and the related changes in the ligand-receptor interactions. To the best of our knowledge, this is the first *in silico* study on the structural and dynamical properties of non-covalent PPARγ antagonists.

## 2. Results and Discussion

### 2.1. Experimental Validation of the Obtained Models and Initial Analyses

The chemical structures and biological data of the studied PPARγ ligands are presented in Table 1 (see Subsection 3.1 in Experimental Section for more details).

The validated docking poses and conformations of the ligands within the receptor obtained from the X-ray complex PDB ID 3VSO were used as starting structures in the MD simulations (see Subsection 3.3. in Experimental Section for more details). Eighteen independent simulations with a length between 100 and 500 ns were executed to describe the phenotypic features of PPARγ ligands. Detailed information about all performed simulations is reported in Table S1 in the Supporting information. Two independent HDX and one NMR studies [1,2,3], along with the available X-ray data, were employed to validate the performed MD simulations with regard to the receptor dynamics and the ligands binding mode reproduction, respectively. The ligands MEKT-21 (PDB ID 3VSO, Table 1) and rosiglitazone (PDB ID 1FM6) were used as references. The PPARγ apo structure was obtained by two subsequent MD runs of total 600 ns after removal of the MEKT-21 ligand from the X-ray complex PDB ID 3VSO.

Based on the root-mean-square-fluctuations (RMSF) data, our MD results agree well with the experimental NMR data (Figure S1). For rosiglitazone (Figure S1A), the MD simulation resembled the NMR data very well and only a small divergence was recorded for the second part of H11 (residues 452–459) due to the perturbations provoked by rosiglitazone fluctuations in this region, which is close to H12. The initial docking solutions were further improved after 100 ns of MD simulations, and the averaged structures overlapped well with those experimentally retrieved for MEKT-21 and rosiglitazone with the root-mean-square deviation (RMSD) of 0.3 and 0.5 Å, respectively. The ligands stabilized the apo form, which was much more flexible than the holo PPARγ, in agreement with experimental data [1,2,3,4]. The fluctuations of several receptor subfragments were greatly reduced, but the level of stabilization was different for the individual compounds (Figure S1B and reference [7]). The part of the H3 region (residues 279–287) was steadier in the presence of either the partial agonist MEKT-21 or the 9p antagonist than the full agonist rosiglitazone, which was in an agreement with HDX and NMR data [1,2,3]. Further, the 4β-sheets (341–351) and H6–H7 (352–377) were also stabilized by the ligands. Notably, the MD simulations also predicted correctly the fluctuation of H12, which was about two times higher for most of the partial agonists and antagonists than for the full agonist. Only rosiglitazone stabilized the activation helix. The same behavior was observed for the loop between H11 and H12 (residues 464–467).

**Table 1:** Structural and activity data of the studied peroxisome proliferator-activated receptor γ (PPARγ) ligands.

No.	Ligand Structure/Name in the Original Source	PDB Complex/Ligand	Effect	Ref.
1	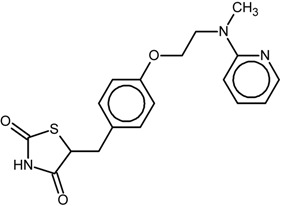	1FM6/BRL	Full agonist	[14]
	rosiglitazone			
2	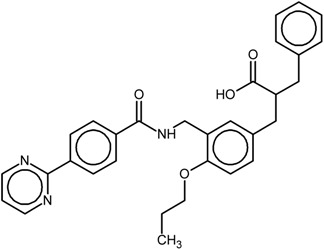	3VSO/EK1	Partial agonist	[15]
	MEKT-21			
3	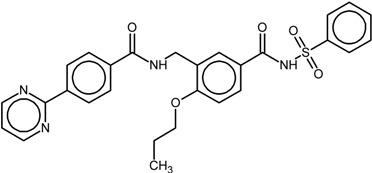	3WMH/JJA	Partial agonist	[16]
	MEKT-75			
4	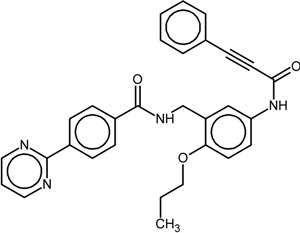	nr *	Antagonist IC_50_ = 174 nM I_max_ = 92%	[7]
	9i			
5	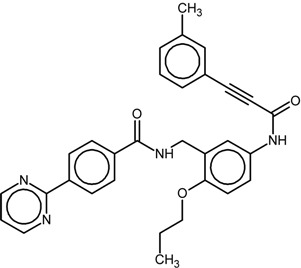	nr	Antagonist IC_50_ = 411 nM I_max_ = 84%	[7]
	9k			
6	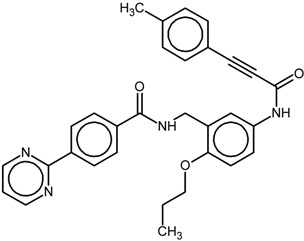	nr	Antagonist IC_50_ = 610 nM I_max_ = 56%	[7]	
	9l			
7	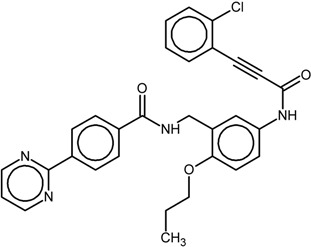	nr	Antagonist IC_50_ = 277 nM I_max_ = 104%	[7]	
	9p			

* nr—Not resolved.

**Figure 1:**
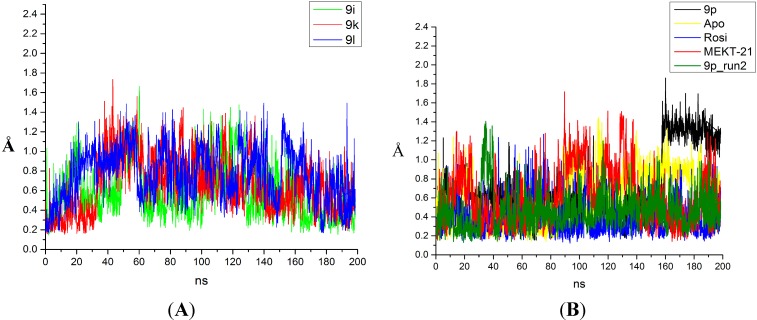
Changes in the root-mean-square-deviations (*Y* axis: root-mean-square deviation (RMSD), Å) of helix 12 in time (*X* axis, ns) in the PPARγ complexes with: (**A**) ligands 9i, 9k, 9l; (**B**) ligands 9p (the two independent molecular dynamics (MD) runs are shown), rosiglitazone (Rosi), MEKT-21 and the PPARγ apo form (Apo).

No significant changes in the dynamics of the ligand-receptor complexes of compounds 9i, 9k, 9l, and 9p have been detected based only on the RMSF analyses (Figure S1B). The similar receptor fluctuations are not unexpected, because the compounds share the same skeleton and differ only in the substituents located close to H12 where more significant changes were observed. This additionally corroborates the good reproducibility of the performed MD runs because no random structural changes were introduced by the ligands. The increased fluctuations of the holo PPARγ receptor in the H11–H12 region (Figure S1B) can be easily explained by the specific binding mode of the studied compounds and agree with the experimental studies [1,2,3,4]. For instance, the unsubstituted phenyl group of ligand 9i had higher mobility whereas the same substituted ring in the compounds 9k, 9l and 9p interacted with and perturbed significantly the residue Leu249, which in turn affected the last part of H11, either stabilizing it or *vice versa*. This is evident from the RMSD plots of the H12 alignment on itself that displays the changes in the activation helix fluctuations during the simulation (Figure 1A,B). The 9p ligand stabilized H12 in a conformation different from the agonist state but also exerted much smaller H12 fluctuations compared to the partial agonist MEKT-21, as well as the 9i and 9l ligands, during the executed independent MD runs. Thus, according to the observed H12 dynamics, the 9i and 9l compounds behave more like partial agonists than antagonists.

The above provided RMSD analysis of H12 also gives an idea about the time necessary for the initial receptor adaptation to the structural changes provoked by the ligands, *i.e.*, for the activation helix to reach a reasonable equilibration. According to the observed RMSD values of H12, most of the complexes reached equilibrium after about 30–60 ns of simulation, depending on the system (Figure 1A,B). Instead, the first part of the simulations represents the adaptation of the activation function-2 (AF-2) region to the introduced changes rather than the real dynamics. For instance, these are the large fluctuations induced by the substituents of the 9k and 9l ligands in H12. After the equilibration period, H12 trapped out of the initial energy minimum, and a reasonable sampling was observed, including also an agonist activation helix conformation. The apo form showed similar behavior, but the activation helix was not able to detach from its initial full agonist position during the first 60 ns, which strongly indicated that partial agonists and antagonists provoke instability of H12 for a short simulation time. Thus, the difference between the apo and holo forms can be easily explained by the ligand perturbations that reshape the H12 conformational landscape. As seen from our results, an equilibration and detachment of the H12 in the apo form was achieved after about 80 ns of simulation time and this has also been confirmed by recently performed MD simulations [4]. However, the mean RMSD of the Cα atoms of H12 in both the apo and the holo receptor were about 0.8–1.0 Å, increasing to 1.2–1.4 Å for the 9p antagonist, respectively. The observed fluctuations (RMSD up to 2.0 Å) indicate that H12 remains close to the LBD in either its apo or antagonist state.

The comparison of the RMSDs of the H12 structure in the apo PPARγ structure and in the complex with 9p showed significant differences (Figure S2). Taking also into account the RMSD data for the remaining ligand-protein complexes (Figure 1A,B), it became evident that both the activation helix conformation and dynamics differed for agonist, partial agonist and full antagonist. Remarkably, after about 150 ns of simulation time during the MD run 1 of the 9p complex, H12 stabilized in a completely new state, which we named here an antagonist conformation. Moreover, diversity in the conformational changes for the apo form was also detected by the above-mentioned RMSD analyses (Figure S2). Although, as already mentioned, no significant changes compared to other nuclear receptors, such as ERα, were observed, this new H12 conformational state led to a dramatic change in the coactivator binding site, which might explain the observed antagonism in view of the receptor-coactivator interactions. However, the analysis of the H12 antagonist state requires a more exhaustive analysis of the conformational landscape, including the principle component analysis, correlation, and clustering, along with convergence analyses by methods like the recently introduced cross-correlations and the Kullback–Leibler divergence (KLD) [17,18] methods, and such studies are currently ongoing in our group. The above analysis, however, provides sufficient evidence for the existence of such an antagonistic state of the PPARγ activation helix. The credibility of this suggestion is further confirmed by the free energy estimation of the studied ligands.

### 2.2. Binding Mode and Free Energies of the Lead Compound 9i

**Figure 2:**
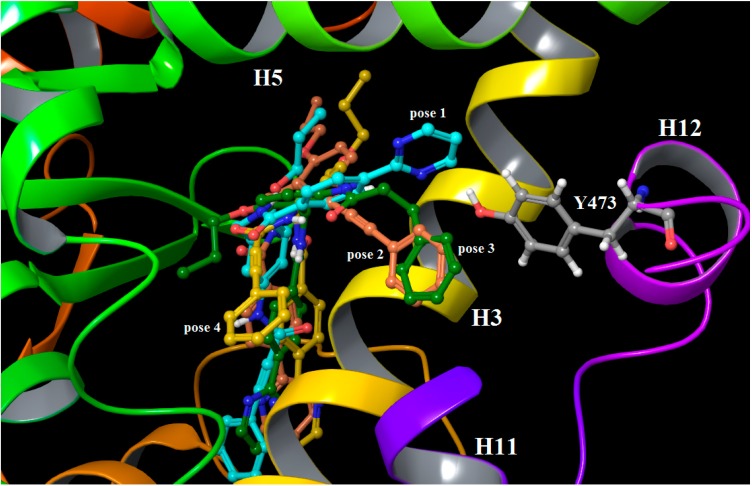
Binding modes of ligand 9i based on the averaged MD structures that correspond to the different input docking poses: pose 1 (cyan), pose 2 (brick), pose 3 (dark green) and pose 4 (dark yellow). The trajectories after 60 ns of simulation time were only used. The ligands and residues are rendered in balls and sticks and the atoms are colored according to their types: C—grey, H—white, O—red, N—blue.

The possible binding modes of the studied ligands were carefully examined because it was shown that for this class of structurally similar compounds a different orientation in the receptor binding site was possible [7]. The X-ray data for MEKT-21 and MEKT-75 partial agonists, which share a very similar scaffold to the antagonists studied here (Table 1), showed a different binding mode and, in particular, dissimilar positions of the common Phe-O-(CH_3_)_3_ group, as observed in their X-ray structures, PDB ID 3VSO and 3WMH, respectively. The picture is even more complex because it is known that, at least in PPARγ, partial agonists have multiple binding modes [3]. Thus, it is reasonable to suggest that, as in the case of partial agonists, the free energy estimated by our MD simulations can represent different binding conformations rather than a single one. Based on these facts, four different docking conformations of the unsubstituted lead compound 9i were initially considered as inputs to the MD runs, and each of them was executed in two independent simulations, named here poses 1 to 4. The conformation in which Tyr473 of H12 interacted with the pyrimidine ring (pose 1) instead of with the phenyl one (pose 2) was about 3 kcal/mol energetically less favorable, according to both molecular mechanics Poisson–Boltzmann Surface Area (MM-PBSA) and molecular mechanics/Generalized Born Surface Area (MM-GBSA) calculations ( Table S2A,B). Although such mirror-like binding mode was observed in other partial agonists [3], we rejected this binding mode as unlikely due to the big energy difference to pose 2 (Figure 2 and Figure S3). The possible binding mode 4 was also unacceptable due to the higher free enthalpic energy and the significantly decreased (about 2-fold) electrostatic contribution. Further, similar MM/PBSA results were obtained for the binding modes 2 and 3 where the differences were mainly in the conformation of the Phe-O-(CH_3_)_3_ group, which was docked toward Met348, *i.e.*, similarly to the MEKT-21 derivative. However, the free energy obtained by MM/GBSA was about 3 kcal/mol lower in pose 2 compared to pose 3. It is broadly thought that the MM/GBSA approach is better for free energy ranking, whereas the MM/MBSA gives more reasonable absolute values [19]; thus, for this analysis the results of the first method are more reasonable. Moreover, in addition to the energetically more favorable energy of pose 2, its phenyl ring was suited in a position that displaced significantly Tyr473 and changed the overall H12 conformation in an outward-facing direction. This effect was observed during the two independent 100 ns long MD runs for each of these two poses. Thus, we cannot exclude pose 3 as an alternative binding mode, but we consider it less probable.

Taking together the overall data of the free energy calculations of 9i ligand, it becomes evident that pose 2 is the most likely one in the LBD pocket. Thus, in all further analyses, including the substituted compounds in the series, only the binding mode with a phenyl ring toward Tyr473 was chosen, as was also presumed previously [7].

### 2.3. Binding Mode, Mechanism of Action and Free Energies of the Selected Partial Agonists and Full Antagonists

Further, we investigated the binding modes and the free energies of binding for the remaining compounds in the series 9p, 9k and 9l (Table 1). For rosiglitazone and MEKT-21 the detected ligand-receptor contacts were almost identical to those observed by X-ray analyses (data not shown).

**Figure 3:**
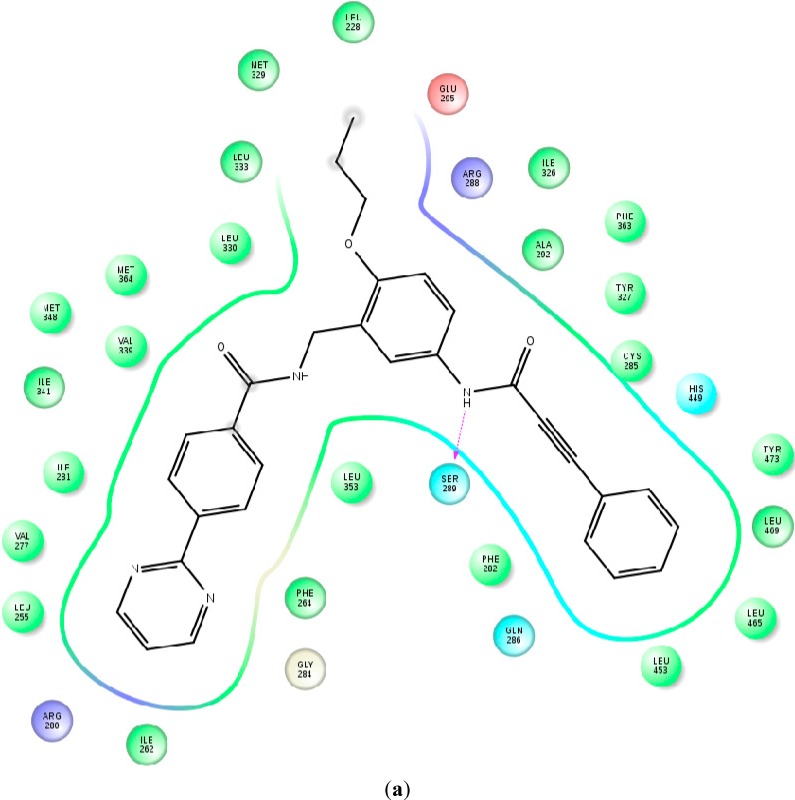

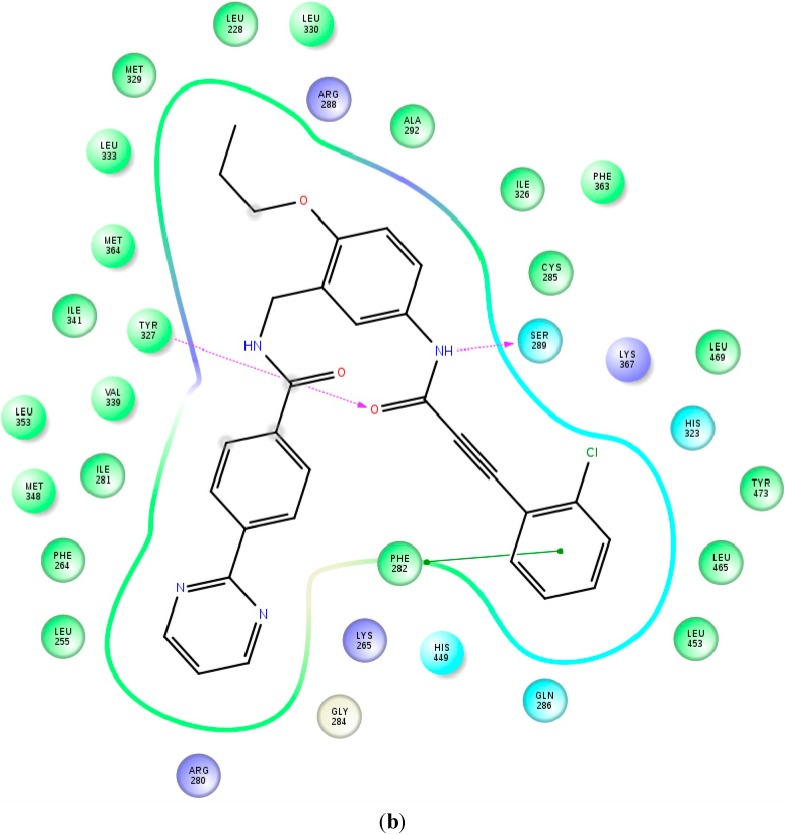
Identified ligand-protein contacts and interactions based on the averaged MD structures of ligand 9i (**a**) and ligand 9p (**b**). The PPARγ residues are colored as follows: charged negative (red), charged positive (violet) hydrophobic (green), glycine (white) and polar (cyan). Solvent exposed areas of the ligands are noted as gray spheres (a detailed legend of the contacts and interactions is given in Figure S4).

Despite the structural similarity, the selected compounds showed some differences in the binding mode (Figure 3 and Figure S4). These differences were partly due to the different Phe-O-(CH3)_3_ group position in LBD and mainly to the decreased flexibility of the substituted phenyl ring. The amide group in antagonist 9p made H-bonds with Tyr327 (the backbone =O) and Ser289 (–NH–). An additional π–π staking of the ligand phenyl ring with Phe282 was also observed. During the pair of independent simulations of ligand 9p, two different orientations of Tyr473 were detected—one in a position toward H4 and one toward H11. Notably, the 9k ligand, for which no transcriptional activity was experimentally available, showed the same behavior. The remaining ligands exhibited only a Tyr473 displacement toward H4, which made 9p and 9k ligands unique and indicated that 9k behaved more like an antagonist. This might explain why only the second and third position substitutions in the phenyl ring transform the compounds into full antagonists.

Further, from Figure S4 it is evident that Tyr327 does not make a hydrogen bond with the ligand’s amide oxygen in either 9k or 9l ligands. Notably, a lesser H12 displacement was observed in the 9l complex due to the stabilization of the methyl group in the 4th position, which makes contacts with H11 and the loop between H11 and H12. Instead, the unsubstituted ring of 9i was much more flexible in the binding pocket moving between the Phe282 and the Tyr473 residues. Moreover, the ligand 9l does not alter the Tyr473 position, probably also due to the increased π–π interactions with this residue. In addition, π–π staking was also observed between Phe264 and the pyrimidine ring accompanied by stronger interactions near the H3 and β-sheets region.

### 2.4. Contributions of the Individual Residues to the Free Energy of Binding/Mechanism of Action

**Figure 4:**
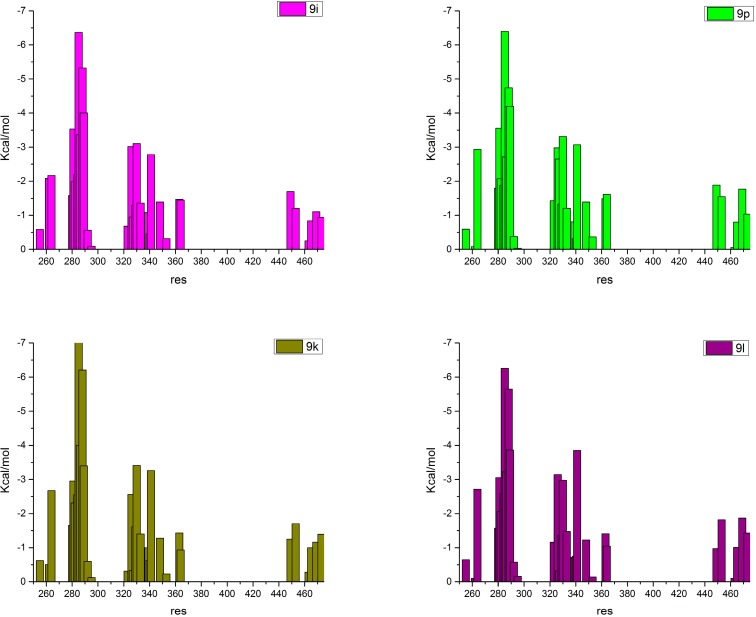
Observed free energy of binding (*Y* axis, kcal/mol) of the individual PPARγ residues (*X* axis, residue number) obtained by the decomposition method for: ligand 9i (magenta), ligand 9p (green); ligand 9k (tobacco-green) and ligand 9l (violet).

To retrieve detailed information of the ligands-receptor interactions and, in particular, the individual residues’ contribution, the free energy decomposition approach was applied. Figure 4 and Figure S5 represent the calculated interactions of the ligands and their free energy of binding to the individual LBD residues. The full agonist rosiglitazone displayed much stronger interactions with H12 and, in particular, with Tyr473 than the partial agonist MEKT-21, in an agreement with experimental data [1,2,3,4]. Instead, the binding of MEKT-21 with residue regions 255–265 (H2′), 279–292 (H3), 320–365 (H5, 1–4 β sheets, H6/H7) and 449–450 (H11) was much stronger compared to rosiglitazone, and this is typical for a partial agonist [1,2]. These data demonstrate at molecular level why the above ligands have different activation pathways and agree well with both NMR and HDX results [1,2,3,4]. This is also an example, along with all the MD results in this study, how the ligand dynamical properties and interactions, obtained by either experimental or theoretical techniques, can be used in the mechanism of action classification. Such a classification approach has been applied to other nuclear receptors to determine the pharmacological profiles ranging from full agonists to full antagonists [20].

The strongest ligand-residue interactions were those with Cys285, observed for all ligands, with an enthalpic free energy of about −6 to −7 kcal/mol. The selected ligands showed a lot of similarity in the interactions, as could be expected considering the similar skeleton shared, but there were some important differences as well. The ligands’ free energy of binding to the individual receptor LBD residues, related to the formation of the coactivator complex, was dissimilar, thus affecting the stabilization of this region, which is important for the whole PPARγ function. For instance, the partial agonist MEKT-21 binds stronger than the antagonist 9p to the 1–4 β-sheets and H5/H6 but much weaker to both H4 and H12, which, along with H3, form the coactivator pocket (Figure 4 and Figure S5). The binding of rosiglitazone, MEKT-21 and 9p to Tyr473 of H12 was 2.2, 0.7 and 1.1 kcal/mol, respectively. All 9i, 9k, 9l and 9p ligands had decreased binding capacity to His449 but increased interactions with the Tyr473 of H12. Diversity in the interactions with H3 residues was also observed. Thus, the results suggest a distinguished binding mode and, thereupon, a mechanism of action between the agonists and the studied series of compounds.

According to the decomposition analysis, the enthalpic free energies of binding to the above LBD regions were almost the same for all the compounds in the series and were equal to about −60 kcal/mol. However, different ligand interactions with the individual residues were observed, which revealed in more details the differences in the mechanism of action of the selected antagonists and their phenotype (Figure 4 and Figure S5, Table S3). These dissimilarities are mainly due to the ligand-residue contacts in the two protein regions of importance for the ligand binding, H3/H11 and H12, respectively. The interactions in these “hot spot” regions also constitute the observed flexibility of the substituted phenyl ring and, consequently, the dynamical properties of the compounds.

All ligands interact with H3, the final and more flexible part of H11, in particular Leu453, and the loop between H11 and H12. These interactions provoke a high flexibility of the phenyl ring, which, in turn, hampers the possibility for H12 to be stabilized in a new, uniform, energetically stable state but still significantly perturbs the activation helix. The mechanism of this process can be easily explained based on the obtained free energy estimation results. The studied compounds cannot form an H-bond with His449, but they bind to Leu453 with the same or even lower free energy than to the above mentioned histidine. This is more evident for the ligands 9i and 9l. The last compounds bind to H3 with about 2 kcal/mol less than 9p and 9k, stabilizing it, and also exhibit higher free energy of interactions with H11 (Figure 4 and Table S3). Indeed, this itself would disturb the agonistic activity of the ligands and explain the observed higher mobility of ligands 9i and 9l, but it is also a strong indicator for their transcriptional activity. Based on the above results, we predict different dynamical properties of 9p, 9k, 9i and 9l ligands. One can expect that 9i and 9l, along with MEKT-21, would be much more dynamically unstable in the AF-2 region than 9k and 9p, and, thereupon, their transcriptional activity might be expected to be different too. This is supported by the experimental results for the compounds MEKT-21, 9i and 9p [7]. While MEKT-21 is a well-defined partial agonist, the 9i exhibited about 10% transcriptional activity, and its LBD complex was inefficient in recruiting either the coactivator or the corepressor [7]. The last can be easily explained by our results: the observed instability in the interactions at AF-2 region for ligand 9i will not lead to a stable H12 position, as is evident from the observed RMSD deviations (Figure 1A,B); thus, neither the coactivator nor the corepressor would be stabilized.

**Figure 5:**
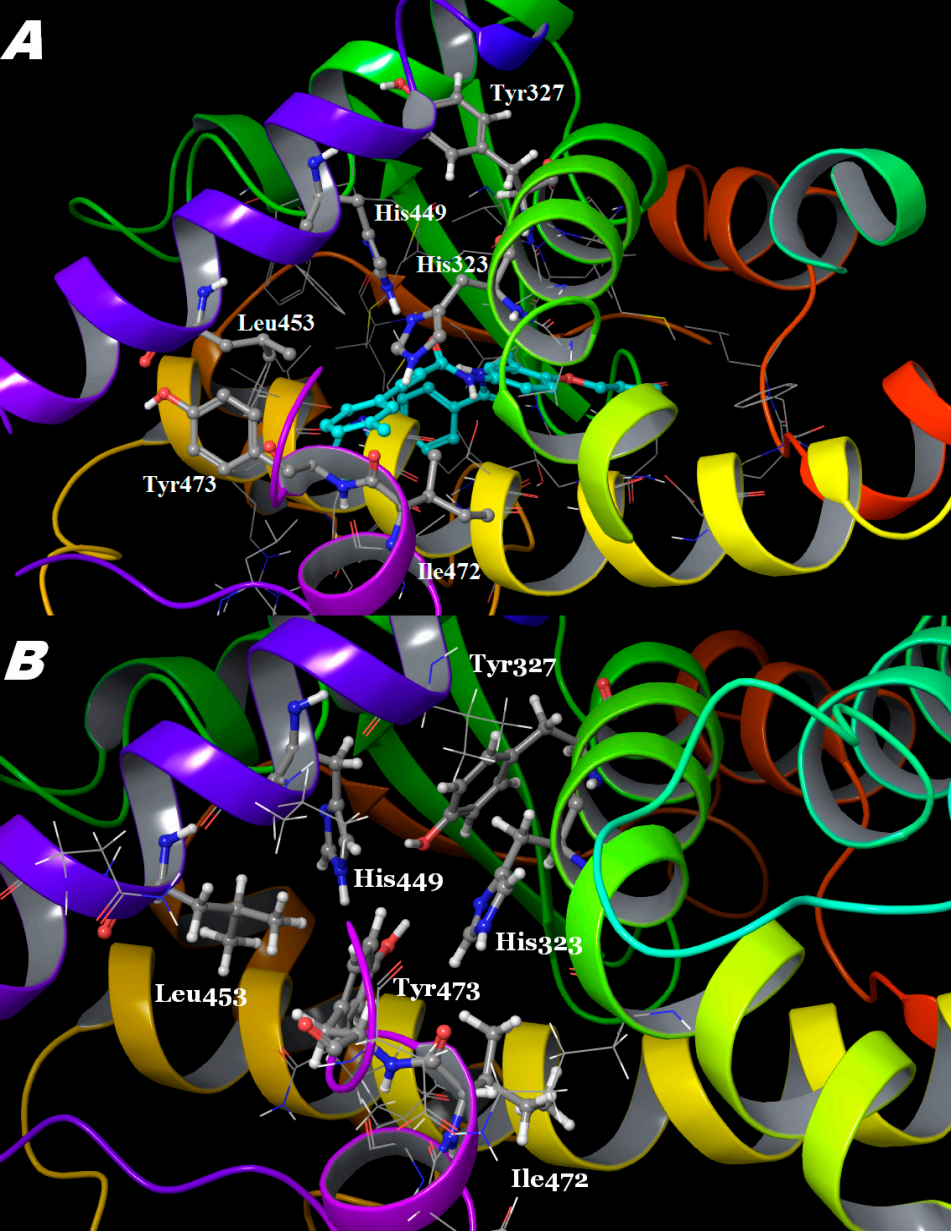
Observed organization of the PPARγ ligand-binding pocket based on the averaged MD structures of: (**A**) the 9k complex; (**B**) the apo form (refer to Figure 2 for the colors).

Next, different direct interactions with H12 have been observed among the studied compounds (Figure 4 and Figure 5, Table S3). Ligand 9k interacts with H12 in a similar way to 9p, and, based on this result, one can expect a very low transcriptional activity. The antagonistic features of these two ligands are linked to Tyr473, which was displaced, and the whole H-bonds network between the two pairs of histidine’s and tyrosine’s residues, observed typically in the apo form, was destroyed. However, the Tyr473 rotation in 9k was not due to the direct ligand-tyrosine contact, as in the case of 9p, but to the strong interactions (−1.2 kcal/mol) between the methyl group at the 3rd position and Ile472 (Figure 5A). It resulted in the displacement of this residue and the whole H12, stabilized Tyr473 in a position toward H11 but also affected the charge clamp residue Glu471, which shifted its position in the same direction (Figure S6). We observed that Lys319 played a significant role in this process too, and the distance between Lys319 and Glus471 increased from 4.6 Å, as seen in PDB ID 3VSO, to 7.3 Å. A detailed study about how the Lys319–Glu471 pair affects the coactivator binding is currently in progress. The significance of the Lys319 point mutations for the PPARγ activity was previously detected [21]. Another example is ligand 9l, which interacted most strongly to H12 but did not affect either the charge clamp or the H-bond network seen in the apo form, and, consequently, exhibited lower inhibition potently [7]. This further supports our hypothesis that ligand 9l could also be expected to have increased transcriptional activity compared to 9k. It is well known that the conformation of the so called charge-clamp pair [10,22], which consists of the residues Lys301 and Glu471, is critical for the coactivator binding [23,24], in parallel with the important role of Tyr473 for the PPARγ agonistic features [5]. Thus, our data reveal the main reason of the observed antagonism of the compound 9p: it can be related to the disruption of the binding pockets of both the natural ligand and the coactivator, due to the described above structural perturbation induced by its interactions. Our results also demonstrate that the interaction with H12 cannot be trivially explained by one residue contribution, *i.e.*, Ty473 displacement, but it is rather due to a more complex network of interactions. An example of such a complex behavior is ligand 9k, the phenotype of which is presumably more related to the antagonists, but it still shows some partial agonistic activity.

Finally, some previous *in silico* studies speculate that the partial agonists change the distance between charge clamp residues, affecting the coactivator binding pocket [25]. Our results demonstrate that for either the agonist, the antagonist or the apo form, this distance remains unchangeable (20.5 ± 1.1 Å), during all the performed simulations. This is consistent with the crystallographic data that show no difference between the apo form, the full and the partial agonists’ complexes [25]. This result additionally supports our conclusion that, at least for the studied complexes, other factors, such as the orientation of Glu471, are crucial for the changes in the coactivator binding pocket, and, consequently, for the observed PPARγ antagonism.

## 3. Experimental Section

The following software was used for calculation, simulation and visualization of the results: MOE (v.2014.091) [26], GOLD (v.5.2) [27], academic version of Maestro Schrodinger (v.10.1) [28], Amber 14 suite [29,30,31].

### 3.1. Structural and Activity Data

The data of the studied PPARγ ligands are presented in Table 1. For the docking study all compounds were built in MOE [26], and energetically minimized conformations were generated, using the MMFF94x force field, with a maximum number of conformers set to 250. The complex of PPARγ with the ligand MEKT-21 (PDB ID 3VSO) was used as a basic structure in the study due to the fact that MEKT-21 was used as a template for the design of the studied PPARγ antagonists [7]. The same receptor structure was also utilized to generate the apo form of the receptor as described in more details below.

### 3.2. Protein Preparation

The MOE tool “Structure preparation” [26] was used to position the missing hydrogen atoms and to assign the correct ionization states of the polar residues in the used X-ray PPARγ structures. The following entities were addressed: the rotamers of –SH, –OH, –CH_3_ and –NH_3_ groups in the residues Cys, Ser, Tyr, Thr, Met, Lys; the ionization states of acids and bases in Arg, Asp, Glu, Lys, His; the tautomers of imidazoles (His) and carboxylic acids (Asp, Glu); the element identities in His and the terminal amides (Asn, Gln). The tool used the generalized Born/volume integral electrostatics model to optimize the titration free energies of all titratable groups. The following parameters were set: temperature 310 K; pH = 7.4; ion concentration: 0.152 mol/L.

### 3.3. Docking

The GOLD (Genetic Optimisation for Ligand Docking) software [27] was used to generate reasonable poses and conformations of the PPARγ ligands into the receptor binding site for the subsequent MD simulations. The GOLD fitness function is made up of four components: protein-ligand HB energy (external H-bond); protein-ligand van der Waals (vdW) energy (external); ligand internal vdW energy (internal); and ligand torsional strain energy (internal torsion). The default GOLD values for the fitness function were used. The docking poses for each of the studied ligands were scored according to the GoldScore scoring function. The first 3–5 best poses (with the highest GoldScore scores) were selected for the subsequent analysis. The docking protocol was validated by docking the ligands with available X-ray structures in the X-ray complex of PPARγ with MEKT-21 (PDB ID 3VSO). We were able to successfully regenerate not only the pose of the original ligand but also of rosiglitazone (PDB ID 1FM6) and MEKT-75 (PDB ID 3WMH). The GOLD docking poses of rosiglitazone were also compared to the poses generated in MOE by a pharmacophore-based docking, using the previously developed pharmacophore model of PPARγ full agonists [32]. The GOLD poses were very similar to those obtained by MOE docking and resembled very well both the orientation in the pocket and the bioactive conformation of the studied ligands in their X-ray complexes. Thus, we concluded that the generated by docking conformations of the studied ligands within the 3VSO receptor site represent reasonable starting structures to be used in the subsequent MD simulations.

### 3.4. Molecular Dynamics

Conventional molecular dynamics (cMD) was carried out, using the Amber 14 suite of programs and the Amber14SB force field [29,30,31]. A truncated octahedral of TIP3P water molecules, 12 Å dimension in each direction and counterions were added to obtain the final solvated system, which consisted of nearly 50,000 atoms. Initially, the systems were energy-minimized in two steps. First, only the water molecules and ions were minimized in 6000 steps while keeping the protein structure restricted by weak harmonic constrains of 2 kcal·mol^−1^·Å^−2^. Second, a 6000 steps minimization with the conjugate gradient method (convergence criterion of 0.1 kcal·mol^−1^·Å^−2^) on the whole system was performed. Furthermore, the simulated systems were gradually heated from 0 to 310 K for 50 ps (NVT ensemble) and equilibrated for 3 ns (NPT ensemble). The production runs were performed at 310 K in a NPT ensemble. Temperature regulation was done using a Langevin thermostat with collision frequency of 2 ps^−1^. The time step of the simulations was 2 fs with a nonbonded cutoff of 9 Å using the SHAKE algorithm [33] and the particle-mesh Ewald method [34]. The convergence analysis of both the individual simulations and the repeated simulations was performed by cross-correlations and KLD methods [17,18,35] to demonstrate that a reasonable sampling was performed even on the flexible receptor parts (data not shown). The averaged structures of two independent simulations were used. To trace out how H12 changes its conformation due to the ligands binding, the root-mean-square-deviations (RMSD) and the root-mean-square-fluctuations (RMSF) were calculated by aligning H12 to its own backbone atoms instead of using the whole protein structure. This is a more precise analysis of the internal H12 structural changes because the flexibility of the long loop between H11 and H12 is difficult to be sampled.

### 3.5. Free Energy Estimation

The free energy estimation was performed by the molecular mechanics Poisson-Boltzmann surface area (MM-PBSA) method using the MMPBSA.py script included in the AmberTools 14 package [29,36]. In order to evaluate the individual contributions of the selected residues, a decomposition of the free energy contributions approach was employed. Pairwise per-residue basis decomposition for the selected protein residues was chosen. Pairwise decomposition calculates the interaction energy between all pairs of residues in the system. All of the decomposed energies were calculated by the MM-GBSA method, whereas the total enthalpic free energies were calculated by both MM-PBSA and MM-GBSA, which differ in the solvation energy assessment. Previously, we used successfully both of these methods during the virtual screening and description of the ligand-receptor interactions in the PPARγ [37]. The MM-PBSA is a widely used approach for free energy calculations, and a detailed description of the methodology was described previously [38,39]. Shortly, for each frame extracted from the MD trajectory (a 50 ps interval was set), the enthalpic free energy was calculated as follows:

(1) $${∆G}_{bind}={∆G}_{gas}+{∆G}_{solv}-T∆S$$

(2) $${∆G}_{gas}={∆G}_{ele}+{∆G}_{vdw}$$

(3) $${∆G}_{solv}={∆G}_{PB}+{∆G}_{nonpolar}$$

(4) $${∆G}_{nonpolar}=\gamma A+b$$

It included vdW contributions, electrostatic contributions, as well as polar and nonpolar contributions to the solvation free energy (ΔG_solv_). The gas-phase free energy (ΔG_gas_) was obtained using the sander module of Amber 14, and the estimation of the polar solvation term ΔG_PB_ was conducted by MM-PBSA.py. ΔG_nonpolar_ was determined from Equation 4, in which A is the solvent-accessible surface area, estimated by using the Molsurf program (a part of the Amber 12 suite of programs) with a solvent probe radius of 1.4 Å, and γ and b are empirical constants set to 0.0072 kcal·mol^−1^·Å^−2^ and 0.92 kcal/mol, respectively. The entropy term (TΔS) was neglected in these calculations. As far as decomposition is concerned, a reasonable way involved splitting the electrostatic contribution to the free energy (ΔG_PB_) into two parts, a self/desolvation energy and an interaction/pairwise energy. The self-energy is the value of ΔG_PB_ when only the residue of interest is charged, while all other residues remain neutral; the interaction energy is the charging energy of the residue in the field of all other charged residues. Thus, the procedure applied here involves two numerical solutions of the Poisson-Boltzmann equation with differently charged states for each residue of interest. The combined trajectories of two independent simulations for each compound and the PPARγ apo form were used for the performed analyses.

## 4. Conclusions

In this study, for the first time, the binding mode and dynamics of a series of non-covalent PPARγ antagonists have been elucidated and the mechanism of their antagonism has been suggested. The PPARγ antagonism has been described with regard to the antagonist-receptor interactions, in particular with the activation helix H12. Initially, the binding mode and dynamic properties of the ligands were revealed. Further, the binding energies of the ligands to both the individual residues and the whole receptor were calculated. Based on these results and those obtained for the apo receptor form, we found that the compounds introduced significant changes in both the PPARγ ligand and coactivator binding pockets, affecting the activation helix H12 conformation. In particular, the H12’s Tyr473 and Glu471 were altered. Considering the well-established role of these residues in the receptor agonistic features and along with the observed dynamical properties of the individual compounds, we concluded that this was the main reason for their PPARγ antagonism. Finally, ranking of the agonistic potential of the compounds with available experimental data was proposed based on the observed ligand-residues interactions and their dynamical properties. There are currently ongoing studies which aim at further elucidation of the structural and dynamical properties of the PPARγ activation helix in its antagonist form.

## Supplementary Materials

Supplementary materials can be found at http://www.mdpi.com/1422-0067/16/07/15405/s1.
