# Supplementary material for: Structural and Dynamical Insight into PPARγ Antagonism: In Silico Study of the Ligand-Receptor Interactions of Non-Covalent Antagonists
Source: Int J Mol Sci. 2015 Jul 8;16(7):15405–24. doi: 10.3390/ijms160715405 (PMC4519905; doi:10.3390/ijms160715405)
Supplement: Supplementary file 1 [file ijms-16-15405-s001.pdf]

Supplementary Information

Table S1. Performed molecular dynamics simulations on the PPARγ apo form and the receptor complexes with the studied ligands.

Simulation Number and System Subjected to MD Runs		Performed MD Simulation Time
1	Apo run 1	100 ns
2	Apo run 2	500 ns
3	9i pose 1 run 1	100 ns
4	9i pose 1 run 2	100 ns
5	9i pose 2 run 1	100 ns
6	9i pose 2 run 2	200 ns
7	9i pose 3 run 1	100 ns
8	9i pose 3 run 2	100 ns
9	9i pose 4 run 1	100 ns
10	9i pose 4 run 2	100 ns
11	9p run 1	200 ns
12	9p run 2	300 ns
13	rosiglitazone	200 ns
14	MEKT-21	200 ns
15	9k run 1	200 ns
16	9k run 2	200 ns
17	9l run 1	200 ns
18	9l run 2	200 ns
	**In total**	3.2 μs

Table S2. (**A**) Enthalpic free energies (kcal/mol) of the studied ligands calculated by the MM/PBSA approach; (**B**) Entalpic free energies calculated by MM/GBSA approach (kcal/mol).

(A)					
**Ligand**	**vdW ***	**Electrostatic**	**E_{PB}**	**E_{POLAR}**	**Total**
9i pose 1	−72.43	−20.62	59.51	−8.39	−41.93
9i pose 2	−73.52	−21.89	57.32	−6.64	−44.73
9i pose 3	−73.95	−25.36	63.13	−8.05	−44.23
9i pose 4	−73.59	**−13.46**	52.65	−8.71	−43.10
9p	−77.44	−26.83	66.38	−8.77	−46.66
9k	−78.33	**−18.59**	56.83	−8.89	−48.98
9l	−77.44	−23.79	61.77	−8.94	−48.39
MEKT-21	−71.06	**−6.68**	33.32	−6.59	−51.01
rosiglitazone	−48.16	−16.16	41.55	−5.78	−28.53
(B)					
**Ligand**	**vdW ***	**Electrostatic**	**E_{GB}**	**E_{SURF}**	**Total**
9i pose 1	−72.43	−20.62	47.77	−9.35	−54.63
9i pose 2	−73.52	−21.89	45.06	−9.44	−59.79
9i pose 3	−73.95	−25.36	52.09	−9.59	−56.88
9i pose 4	−73.59	**−13.46**	41.52	−9.49	−55.00
9p	−77.44	−26.83	50.93	−9.83	−63.16
9k	−78.33	**−18.59**	44.45	−9.83	−62.30
9l	−77.44	−23.79	48.88	−9.75	−62.10

Table S2. *Cont.*

Ligand	vdW *	Electrostatic	EGB	ESURF	Total
MEKT-21	−71.06	**−6.68**	30.82	−9.39	−56.31
rosiglitazone	−48.16	−16.16	34.20	−6.60	−36.69

* Contributions of the individual free energy terms: van der Waals (vdW), Electrostatic, Solvation (E_{GB}), E_{SURF} and Total. The standard errors were in the order of 0.2–0.4 kcal/mol for all energy values and were omitted for simplicity. The higher electrostatic energies observed for some of the compounds are marked in bold (see the text).

Table S3. Computed decomposition free enthalpic energies (kcal/mol) of the identified ligands-residues interactions of the studied compounds.

Residue	Rosiglitaz	MEKT-21	9i	9p	9k	9l
255	−0.08	−0.73	−0.58	−0.59	−0.63	−0.65
262	−0.49	−0.21	−2.08	−0.08	−0.51	−0.1
264	−0.57	−2.73	−2.17	−2.94	−2.67	−2.72
280	−0.18	−1.75	−1.58	−1.8	−1.65	−1.57
281	−1.37	−3.67	−3.53	−3.55	−2.95	−3.06
282	−0.85	−2.32	−1.99	−2.07	−2.31	−2.06
284	−1.93	−3.12	−2.19	−2.58	−2.55	−2.59
285	−6.0	−7.77	−6.36	−6.40	−7.26	−6.26
286	−2.46	−3.52	−3.36	−2.71	−4.0	−3.23
288	−2.04	−3.02	−5.32	−6.14	−6.21	−5.65
289	−2.71	−7.0	−4.0	−4.2	−3.4	−3.87
292	−0.08	−0.12	−0.56	−0.38	−0.6	−0.58
295	−0.01	0.046	−0.09	−0.03	−0.12	−0.16
323	−0.9	−1.0	−0.68	−1.43	−0.31	−1.16
326	−2.08	−1.66	−3.02	−2.98	−2.56	−3.15
327	−2.24	−3.7	−0.95	−2.65	−0.32	−0.31
329	−0.11	−0.12	−1.3	−1.33	−1.61	−1.36
330	−2.51	−2.79	−3.1	−3.31	−3.41	−2.97
333	−0.34	−0.25	−1.36	−1.2	−1.4	−1.48
339	−1.26	−1.73	−1.08	−0.81	−1.0	−0.71
340	−0.5	−0.46	−0.46	−0.31	−0.63	−0.75
341	−2.98	−4.92	−2.78	−3.07	−3.26	−3.85
348	−1.01	−1.87	−1.39	−1.39	−1.28	−1.22
353	−0.57	−1.12	−0.32	−0.37	−0.23	−0.14
363	−1.49	−1.81	−1.47	−1.49	−1.43	−1.41
364	−1.55	−1.87	−1.45	−1.62	−0.93	−1.04
449	−2.21	−2.43	−1.7	−1.89	−1.45	−0.97
453	−0.41	−1.03	−1.2	−1.55	−1.71	−1.82
463	−0.04	−0.89	−0.25	−0.05	−0.28	−0.1
465	−0.25	−0.64	−0.84	−0.8	−1.0	−1.01
469	−0.71	−0.92	−1.1	−1.77	−1.16	−1.87
473	−2.18	−0.75	−0.94	−1.03	−1.39	−1.42

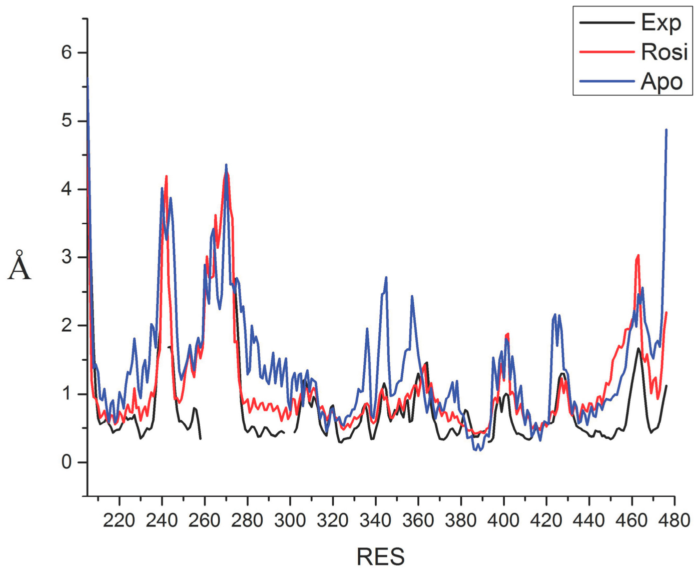

(**A**)

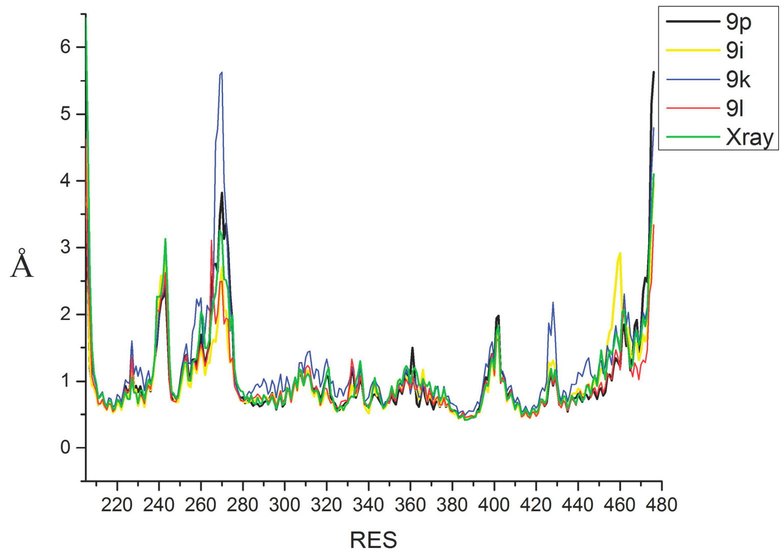

(**B**)

Figure S1. Changes in the observed root-mean-square fluctuations (*Y* axis, RMSF Å) of PPARγ residues (*X* axis, residue number) by the MD simulation employed on: (**A**) rosiglitazone (red), experimental NMR (black) and the Apo form (blue); (**B**) Partial agonist and antagonists. Note that for some receptor regions the NMR data are not available.

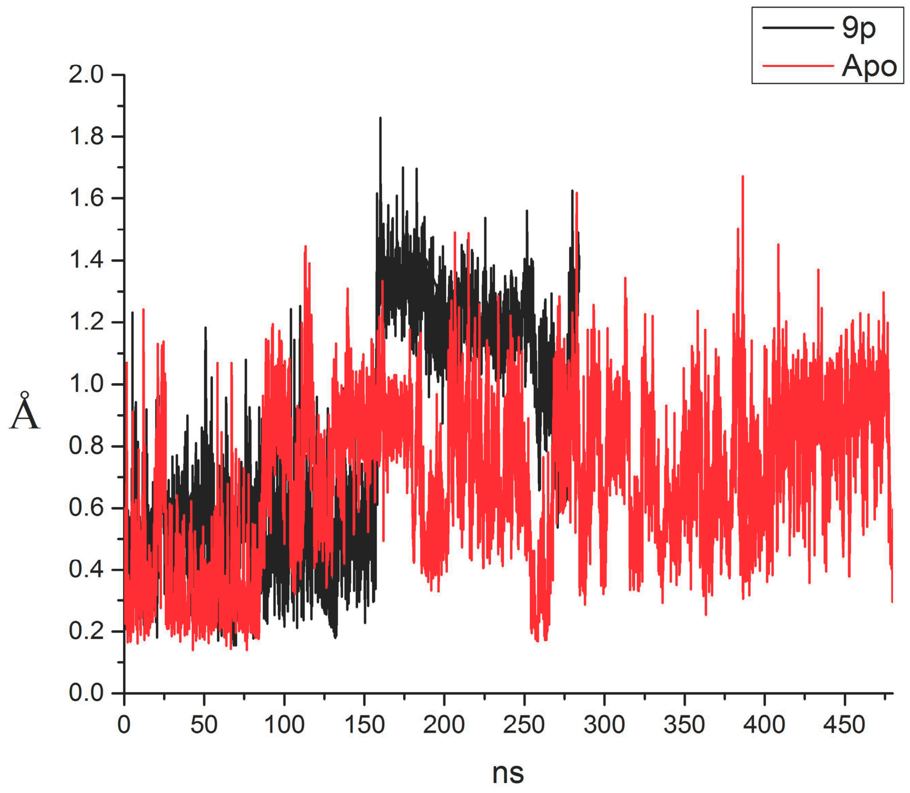

Figure S2. Changes in the observed root-mean-square deviations (*Y* axis: RMSD, Å) of helix H12 in time (*X* axis, ns) during the first MD run of ligand 9p (in black), which took 300 ns of simulation time, and the apo receptor form (in red; 500 ns in length). Note that the H12 agonistic conformation was also sampled in the apo form for the period between 250 and 270 ns of the simulation time.

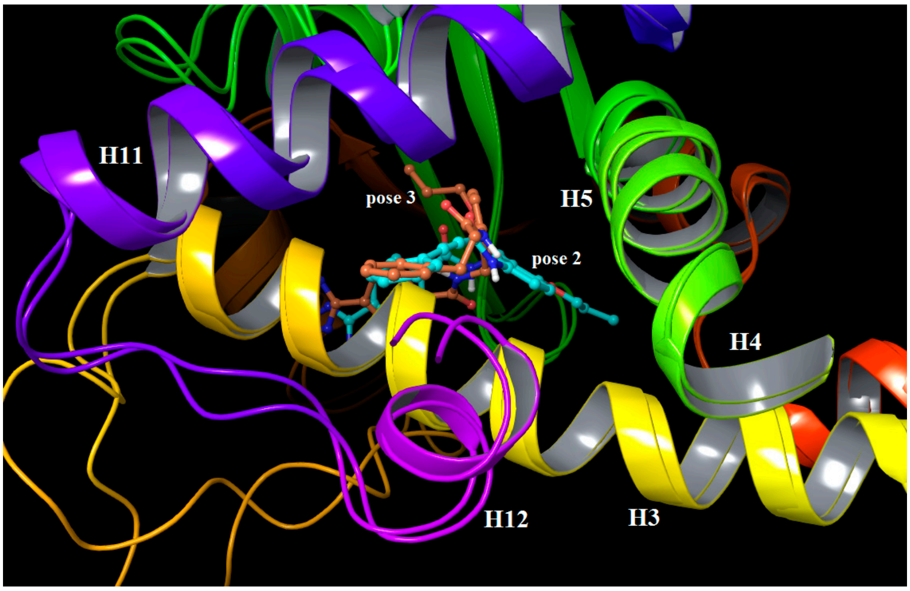

Figure S3. Binding poses 2 and 3 of ligand 9i based on the averaged structures obtained, which correspond to the different input docking poses. The trajectories after 60 ns of simulation time were only used. The ligands are rendered in balls and sticks and the atoms are colored according to their types: C—grey, H—white, O—red, N—blue.

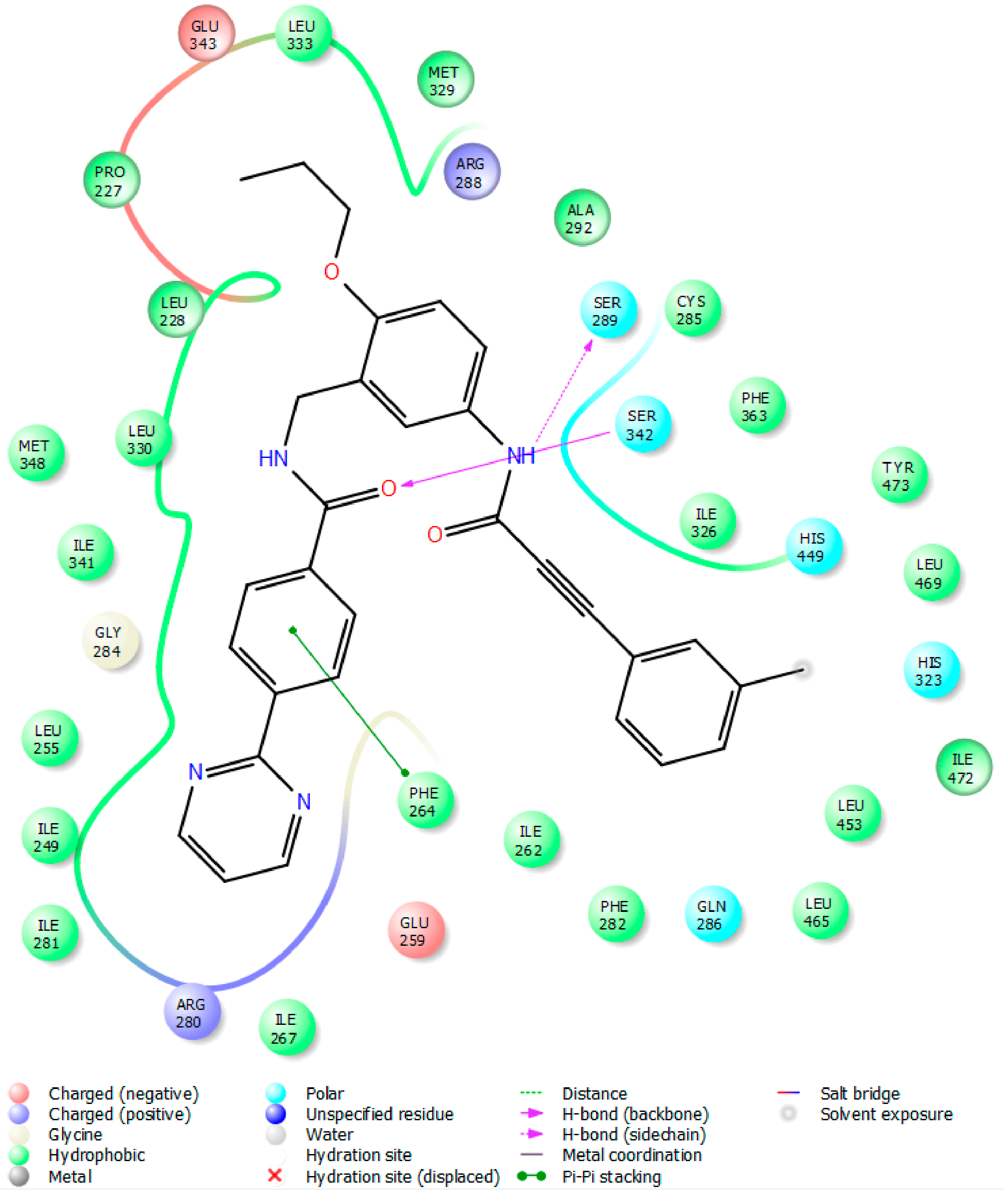

(a)

Figure S4. *Cont.*

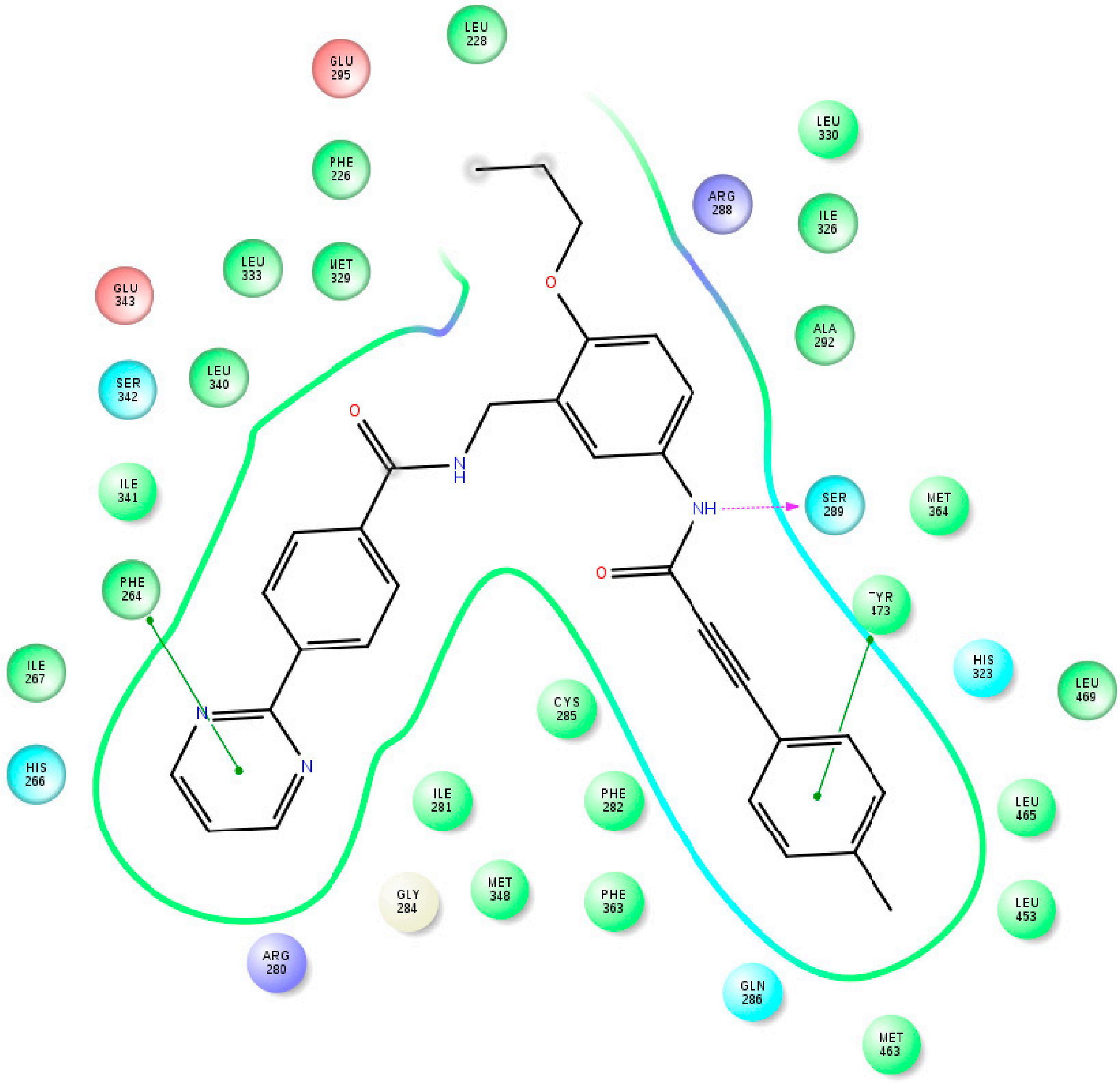

(**b**)

Figure S4. Identified ligand-protein contacts and interactions based on the averaged MD structures: ligand 9k (**a**); ligand 9l (**b**). The residues are colored as follow: charged negative (red), charged positive (violet), hydrophobic (green), glycine (white) and polar (cyan). Solvent exposed areas of the ligand are noted as grey spheres. For more details see the legend in (**a**).

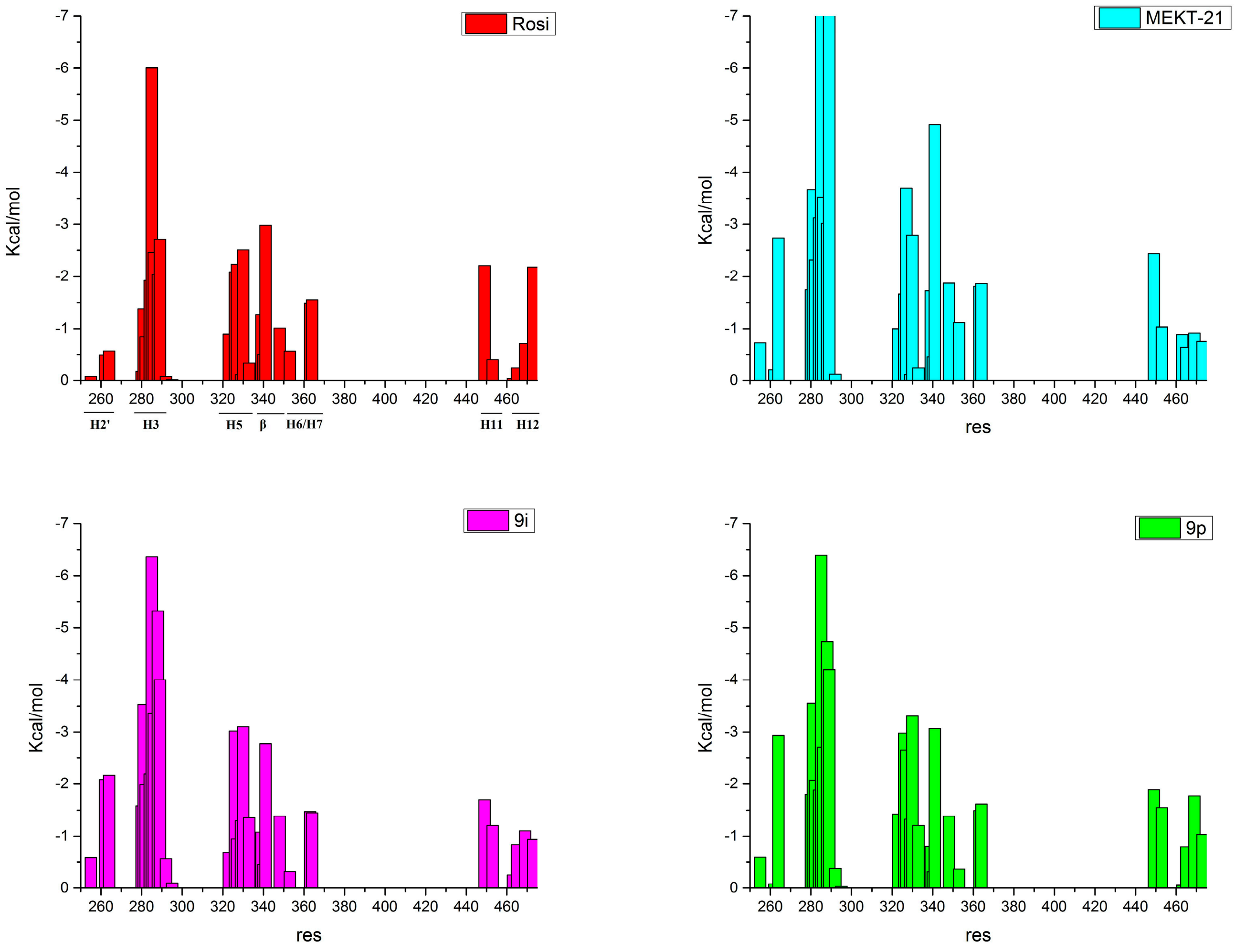

Figure S5. Observed free energy of binding (*Y* axis, kcal/mol) of the individual residues (*X* axis, residue number), obtained by the decomposition method of: rosiglitazone (red), MEKT-21(cyan), ligand 9i (magenta) and ligand 9p (green).

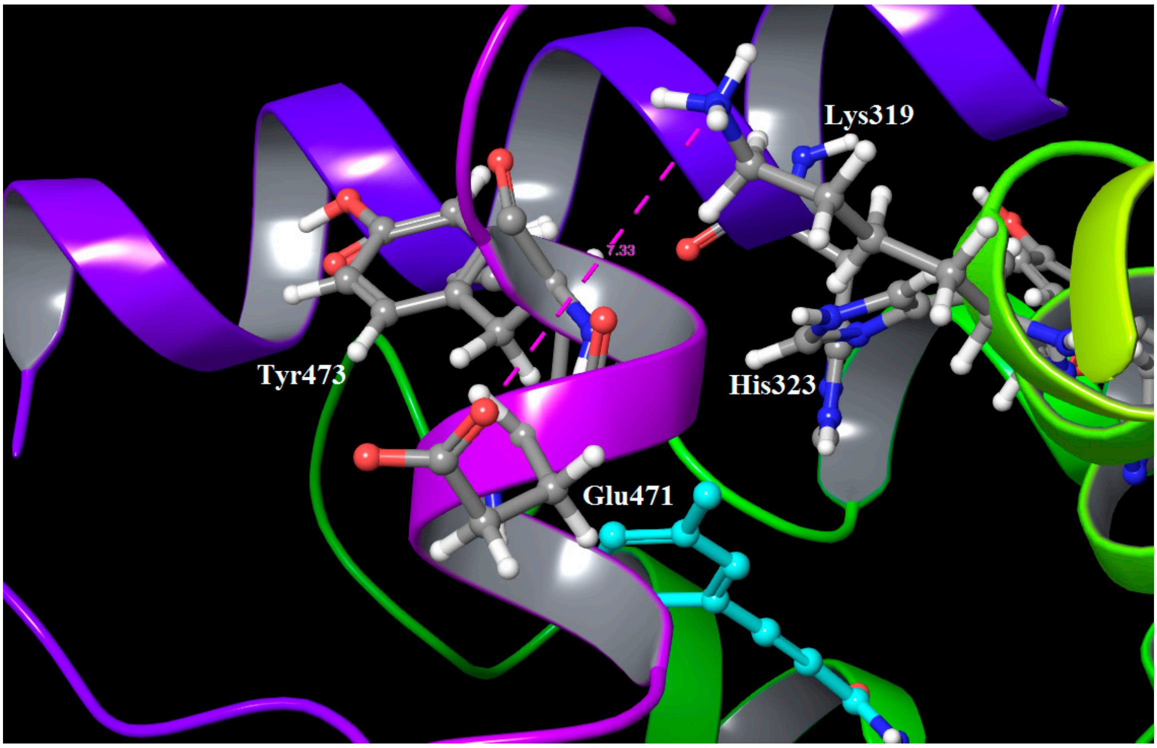

Figure S6. Observed organization of the PPARγ coactivator binding pocket, based on the averaged MD structures in the 9k complex.
